# TGR5: A Novel Target for Weight Maintenance and Glucose Metabolism

**DOI:** 10.1155/2011/853501

**Published:** 2011-06-21

**Authors:** Xiaosong Chen, Guiyu Lou, Zhipeng Meng, Wendong Huang

**Affiliations:** ^1^Division of Gene Regulation and Drug Discovery, Beckman Research Institute, City of Hope National Medical Center, 1500 East Duarte Road, Duarte, CA 91010, USA; ^2^Department of Plastic Surgery, Union Hospital of Fujian Medical University, 29 Xinquan Road, Fuzhou, Fujian 350001, China; ^3^Department of Biochemistry and Molecular Biology, Third Military Medical University, Chongqing 400038, China

## Abstract

TGR5, an emerging G protein-coupled receptor, was identified as a membrane receptor for bile acids. The expression of TGR5 and its function are distinct from the previously identified nuclear bile acid receptor, farnesoid X receptor (FXR). These two bile acid receptors complement with each other for maintaining bile acid homeostasis and mediating bile acid signaling. Both receptors are also shown to play roles in regulating inflammation and glucose metabolism. An interesting finding for TGR5 is its role in energy metabolism. The discovery of TGR5 expression in brown adipocyte tissues (BATs) and the recent demonstration of BAT in adult human body suggest a potential approach to combat obesity by targeting TGR5 to increase thermogenesis. We summarize here the latest finding of TGR5 research, especially its role in energy metabolism and glucose homeostasis.

## 1. Introduction

Bile acids (BAs) are the main products of cholesterol catabolism, which constitutes a major route for the elimination of surplus cholesterol. There are four major types of BAs: cholic acid (CA) and chenodeoxycholic acid (CDCA) are primary BAs; deoxycholic acid (DCA) and lithocholic acid (LCA) are secondary BAs, which are converted from primary BAs by bacterial enzymes in the intestine [1]. BA synthesis is restricted to hepatocytes, and there are two distinct pathways of BA synthesis: the classic/neutral and the alternative/acidic pathways [2]. After synthesis, BAs are transported to and stored in the gallbladder. BAs are secreted into the small intestine in response to dietary intake and function to emulsify and facilitate the absorption of dietary lipids and fat-soluble vitamins. However, approximately 95% of BAs are reabsorbed and transported back to the liver from the ileum, the distal part of the small intestine. This system is known as enterohepatic circulation, which provides a relatively constant pool of BAs. The levels of BAs need to be tightly regulated, to control not only cholesterol levels but also the potential toxic effects of BAs when they reach abnormal levels. For example, both genetic diseases and liver toxins may induce higher levels of BAs in the liver, thereby resulting in a liver disease known as cholestasis [3]. 

It is now clear that, in addition to their important roles in nutritional absorption, BAs are signaling molecules that can activate BA receptors to initiate signaling pathways and regulate gene expression. Two major receptors for BAs have been identified: (1) the nuclear receptor, FXR, and (2) the G protein-coupled receptor (TGR5). FXR is a member of the nuclear hormone receptor superfamily. Among the 48 currently identified nuclear hormone receptors, FXR belongs to a subcluster of nuclear receptors that play key roles in metabolic regulation [4]. These receptors work as sensors of metabolic signals and regulate metabolism by changing the expression profiles of essential components in specific metabolic pathways. FXR was first cloned in 1995 [5] and is highly expressed in the liver, intestine, kidney, and adrenal glands. There is now a large body of evidence indicating that a critical function of FXR is to control BA homeostasis in order to prevent BA-induced liver toxicity [6–10]. FXR regulates BA levels by adjusting the expression of a program of essential gene components that are involved in BA synthesis, transportation, conjugation, and detoxification in the route of the enterohepatic circulation of BAs [11–13]. FXR is also an important mediator of BA signaling and regulates diverse physiological functions. For example, activation of FXR induces the expression of genes involved in enteroprotection [14, 15]. Our group and others have identified novel functions of FXR in liver regeneration, hepatic inflammation, and hepatocarcinogenesis [16–19]. 

## 2. TGR5 and Bile Acid Metabolism

TGR5 is a recently identified plasma membrane-bound, G protein-coupled receptor for BAs. TGR5 is encoded by a single exon that maps to chromosome 1C3 in mouse and 2q35 in humans. Homologs of TGR5 can also be identified in aquatic vertebrates, indicating a potentially conserved role of TGR5 during evolution. TGR5 is ubiquitously expressed, but its expression levels vary among different tissues, with high expression in liver, intestine, brown adipose tissue, and spleen [20, 21]. *In vitro* studies have demonstrated that activation of TGR5 transduces signal through Gs protein-mediated cAMP accumulation [22]. The order of potency of different BAs on TGR5 activation is different from FXR, indicating that these two receptors may have differential functions in mediating the effects of BAs (Table 1).

The role of TGR5 in bile acid metabolism was confirmed in TGR5 knockout mice. Total BA pool size in TGR5 knockout mice was significantly decreased by 21–25% compared with that of the wild-type mice, suggesting that TGR5 contributes to BA homeostasis [20]. However, the exact function of TGR5 and the downstream pathways of TGR5 in regulating BA levels are still unclear. Interestingly, TGR5 activation increased the expression of endothelial nitric oxide synthase (eNOS) [23], which may limit hepatotoxicity of BAs as well as lipid peroxidation. This result suggests that, similar to FXR, TGR5 may have liver protection function.

Another interesting study indicating a potential role of TGR5 in BA metabolism is its function in gallstone formation. TGR5 knock-out mice did not develop gallstones when they were fed a lithogenic diet [24]. Furthermore, TGR5 was highly expressed in human gallbladder epithelium at both the mRNA and protein levels, and the mRNA levels of TGR5 were significantly elevated in the presence of gallstones. TGR5 may promote gallstone formation through stimulating chloride and fluid secretion in gallbladder epithelial cells in response to BAs [25]. Recently, TGR5 was found to localize in mouse and human primary cilium of cholangiocytes, indicating a potential role of TGR5 in the biliary diseases [26]. This was further confirmed by a report that TGR5 appeared to be a likely disease gene in the first genome-wide association analysis of primary sclerosing cholangitis (PSC) [27]. A potential role of TGR5 in this disease is that TGR5 may affect cAMP-dependent cholangiocellular HCO_3_ umbrella [28]. In addition, arginine vasopressin (AVP) has been suggested to play a role in bile homeostasis [29]. TGR5 was reported to partially stimulate AVP secretion after two-thirds partial hepatectomy and bile duct ligation, therefore may protect the liver against shear stress and BA overload [26, 30].

In summary, BAs mainly signal through two receptors, FXR and TGR5. FXR is key to controlling the homeostasis of BAs and preventing the toxic effects of BAs on hepatocytes. In contrast, TGR5 may establish a secondary and backup pathway for maintaining BA levels and for preventing BA-induced toxicity. 

## 3. TGR5 and Inflammation

TGR5 mRNA was detected in the resting CD14+ monocytes in fractionated human leukocytes, adherent alveolar macrophage cells [31], and Kupffer cells in liver, indicating a potential role of TGR5 in modulating inflammation. Activation of TGR5 in Kupffer cells [32] and THP-1 cells overexpressing TGR5 [31] suppressed lipopolysaccharide- (LPS-) induced productions of cytokines, suggesting that TGR5 is a mediator in the suppression of macrophage functions by BAs. Treatment of oleanolic acid (OA), a specific and potent TGR5 agonist, before or at the early onset of multiple sclerosis animal model, ameliorated neurological signs of the disease, indicating that TGR5 modulates inflammation and immune responses *in vivo* [33]. Previously, we demonstrated that FXR antagonizes NF-*κ*B-mediated hepatic inflammation [19]. Whether TGR5 plays a similar role in suppressing NF-*κ*B function during hepatic inflammation will be an interesting future study.

## 4. TGR5 and Glucose Metabolism

TGR5 may play a potential role in type 2 diabetes, as suggested by a recent finding that OA treatment lowered serum glucose and insulin levels in mice fed with a high-fat diet. Moreover, OA treatment enhanced glucose tolerance [34]. Activation of TGR5 induced the production of glucagon-like peptide-1 (GLP-1) in an enteroendocrine cell line STC-1 [35], which was further confirmed by a recent report using TGR5 overexpression mice. The authors showed that TGR5-induced intestinal GLP-1 release led to improved function of liver and pancreas and enhanced glucose tolerance in obese mice [36]. However, a recent study suggested that common genetic variation within the TGR5 gene might not play a major role in the development of prediabetic phenotypes in white population at an increased risk for type 2 diabetes mellitus [37]. Therefore, the exact role of TGR5 in glucose metabolism requires more investigation.

## 5. TGR5 and Energy Metabolism

Overweight and obesity are now viewed as chronic diseases in the whole world that threaten public health if not treated [38]. Obesity is the result from imbalance of energy metabolism, in which the energy intake exceeds energy expenditure. It is now known that two types of adipose tissues play opposite roles: white adipose tissue (WAT) stores energy in the form of lipid and brown adipose tissue (BAT) dissipates energy as heat by thermogenesis [39]. The selection of BAT as a site for energy dissipation is because it contains much higher number of mitochondria compared to other tissues. Moreover, the expression of a protein called uncoupling protein 1 (UCP1, thermogenin) in the inner membrane of mitochondria in BAT is high, and this protein is able to help generate heat instead of ATP in mitochondria. The critical role of BAT in energy burning is becoming more and more recognized in rodent models and recently in human also. Three reports published back to back in *New England Journal of Medicine* demonstrate that adult humans have significant amount of metabolically active BAT [40–42]. Is it possible to manipulate BAT to combat obesity? One way for this purpose is to increase the amount of BAT. Endogenous signals and transcription factors are being identified that control BAT differentiation and development [43–45]. Another possibility is to simply transplant BAT. However, both approaches need more studies and it may take longer to develop a technique to increase BAT volume. Another concern will be the potential long-term deleterious effect when you expand the BAT but cannot remove it easily. An alternative way to burn energy is to increase the activity of BAT. Thus, TGR5 ligands are potential drug candidates for this purpose. 

TGR5 is a key factor in energy expenditure by regulating metabolism. Activation of TGR5 by BAs increases energy expenditure in brown adipose tissue, preventing obesity and resistance to insulin [46]. This effect is FXR-independent but TGR5-dependent. TGR5 mediates the induction of the cyclic-AMP-dependent thyroid hormone activating enzyme type 2 iodothyronine deiodinase (D2), which is essential for the effect of BAs. BAs preferentially induce D2 expression in thermogenical tissues of mouse brown fat and human skeletal muscle via a TGR5-dependent manner. D2 subsequently converts thyroxine (T4) to tri-iodothyronine (T3). T3 is predicted to induce uncoupling protein (UCP) expression. UCP is known to dissipate the proton gradient in the electron transport chain. Therefore, the activation of BAs/TGR5/cyclic AMP/D2/T3/UCP pathway causes a decrease in the synthesis of ATP and thus regulates energy homeostasis. A further study on the responses of TGR5 null mice to high-fat diet in part supports this notion. Female TGR5 –/– mice on a high-fat diet gained more body weight than wild-type mice [20].

An interesting question is whether activation of TGR5 by natural or synthetic ligands would reduce body weight caused by the high-fat diet. Oleanolic acid (OA), a natural ligand of TGR5 isolated from *Olea europaea*, indeed abolished the gain of the body weight by the high-fat diet in an animal model [34]. It improved the glucose tolerance and metabolic disorders as well. However, whether the effects of OA are TGR5-dependent still needs to be determined. Nevertheless, the results highlight a potential use of TGR5 agonists in the antidiabetic or antiobesity studies. 

Besides OA, several other types of secondary BAs are also natural ligands for TGR5, for example LCA, TLCA, DCA, CDCA, and CA [47]. However, these ligands are either toxic or not sufficiently safe. Among them, CDCA appears to be a promising ligand and has been applied in clinical practice [48]. Thyroid hormone treatment for weight loss is known to cause side effects in both urinary nitrogen excretion and heart rates, which are not seen in patients treated with BAs [42, 49]. However, a higher dose of CDCA will elevate serum levels of AST and ALT in patients due to liver damage. In this sense, development of natural or semisynthetic TGR5 ligands may be a future direction for clinical trials. One group of the synthetic TGR5 ligands is the semisynthetic steroidal TGR5 agonists, for example 6EMCA, which is a derivative of CDCA. The second groups are the synthetic nonsteroidal TGR5 agonists, which may improve metabolic homeostasis, pancreatic insulin secretion, and inflammation. The synthetic ligands are summarized in the Table 2 [21, 36, 47, 50, 51]. In addition, discovery of new natural compounds as TGR5 agonists would be also a promising future direction. 

## 6. Concluding Remarks 

TGR5 is an emerging membrane receptor for mediating bile acid signaling. Recent studies highlight it as a novel target for regulating glucose and energy metabolism. The TGR5 signaling in BAT and muscle, two important sites of energy dissipation, suggests that it may be a novel target to combat obesity. We anticipate that, in the near future, more potent and specific TGR5 ligands will be identified and go into clinical trials for obesity and diabetes treatments. 

## Figures and Tables

**Table 1:** Comparison between two BA receptors in liver.

Receptor	FXR	TGR5
Ligands*	CDCA > LCA, DCA > CA	LCA > DCA > CDCA > CA
Liver cell expression	Hepatocyte, stellate cell, Sinusoid endothelial cell	Sinusoid endothelial cell Kupffer cell, hepatocyte
Functions in liver	BA metabolism, antiapoptosis Anti-inflammatory response, liver regeneration, HCC suppression	Suppress LPS response, glucagon expression, energy metabolism, liver inflammation, and HCC?

*CDCA: chenodeoxycholic acid; CA: cholic acid; DCA: deoxycholic acid; LCA: lithocholic acid; LPS: lipopolysaccharide.

**Table 2:** List of currently available synthetic TGR5 ligands.

*A: semisynthetic steroidal ligands*	
23(S)-methyl-CA	
23(S)-methyl-CDCA	
6alpha-ethyl-23(S)-methyl-3alpha, 7alpha-dihydroxy-5beta-cholan-24-oic acid	
6alpha-ethyl-23(S)-methyl-CA (INT777)	

*B: nonsteroidal ligands*	
Quinazolinones	
Imidazol[1,2-a][1,2]doazepin	
Quinolines	
BR27	
3-aryl-4-isoxazolecarboxamides	
6-methyl-2-oxo-4-thyiophen-2-yl-1,2,3,4-tetrahydropyrimidine-5-carboxylic acid-benzeyl ester	
